# A teacher's perceptions of the impacts of the Zero Violence Brave Club in students' wellbeing

**DOI:** 10.3934/Neuroscience.2025023

**Published:** 2025-09-28

**Authors:** Marta Soler-Gallart, Aitor Galar, Ane Olabarria, Ane López de Aguileta, Aitor Alzaga, Garazi López de Aguileta, Cristina Pulido, Ramon Flecha

**Affiliations:** 1 Department of Sociology, University of Barcelona, Av/ Diagonal 690, 08034 Barcelona, Spain; 2 Department of Pedagogy, Rovira i Virgili University, Carrer de l'Escorxador, s/n, 43003 Tarragona, Spain; 3 Department of Didactics and School Organisation, University of the Basque Country UPV-EHU, Oñati Plaza 3, 20018 Donostia-San Sebastián, Spain; 4 Department of Journalism and Communication Sciences, Autonomous University of Barcelona, C/ de la Vinya 738, 08913 Bellaterra, Spain

**Keywords:** violence, toxic relations, brain architecture and functioning, Zero Violence Brave Club, neural activity, cognitive and affective schemata

## Abstract

In recent years, there has been increasing focus in neuroscience on the influence of social relationships and experience in the brain. Socioneuroscience has been created to make contributions to fully understand the social relations that influence the architecture and functioning of the brain. The scientific literature has shown the impact of the coercive dominant discourse that promotes violent relationships on neural changes that lead to negative health consequences. However, less research has been conducted on the changes produced when violent relationships are overcome and prevented. This article advances in this direction by analyzing a teacher's perception on the impact of the Zero Violence Brave Club (ZVBC) intervention on his students' wellbeing. To that end, data collection through communicative observation was gathered over five months of the ZVBC implementation in a 2nd grade primary school classroom. Our results indicated that, according to the teacher, relationships among students improved from the very first week throughout the implementation, improving the classroom climate, students' motivation, and wellbeing.

## 1. Introduction

Social relationships and interpersonal experiences play a significant role in shaping the human brain. When these relationships are toxic, the negative consequences for brain functioning and overall health are profound. According to scientific studies, behavioral stress affects the structure and function of the prefrontal cortex [1],[2], a key brain region involved in decision-making, impulse control, emotional regulation, and executive functions. These changes can manifest as a reduced ability to plan, concentrate, or properly manage emotions, especially when the stress is chronic. As another negative consequence, stress also can lead to changes in the epigenome, affecting, as part of a broader set of changes, mood regulation [3],[4].

Additionally, scientific literature has suggested that toxic relationships, such as disdainful hookups, can distort the cognitive and emotional interpretation of autobiographical memories [5]. Under the influence of the coercive dominant discourse that imposes violent relationships and behaviors as the attractive and desirable ones, such relationships are often remembered with feelings of attraction, which contributes to further victimization [6]. Blackburn and Epel [7] found that strained and toxic relationships, in particular, triggered negative thoughts and chronic stress, which in turn contributed to the shortening of telomeres. The quality of our interpersonal relationships, whether supportive or adverse, can significantly modulate physiological functioning by regulating internal molecular processes that are essential for health and biological functioning [8]. Indeed, stress exerts multiple negative effects on gastrointestinal function, as reported in the following research [9]: It causes alterations in intestinal motility, which can manifest as diarrhea or constipation due to irregular muscle contractions; increases visceral perception, heightening sensitivity to abdominal pain, and discomfort; alters gastrointestinal secretions, affecting digestion and mucosal protection; increases intestinal permeability, facilitating the entry of harmful substances, and contributing to inflammation; impairs the regenerative capacity of the intestinal mucosa and reduces local blood flow, hindering tissue repair; and has a negative impact on the intestinal microbiota, disrupting the balance of beneficial bacteria, and promoting dysbiosis, which can worsen gastrointestinal symptoms and affect the person's overall health.

Furthermore, toxic stress during adolescence has a stronger impact on the hypothalamic-pituitary-adrenal (HPA) axis than the same stress experienced in adulthood [10]–[12]. Another scientific study on biological manifestations of toxic stress states that it increases the likelihood for morbidity and premature mortality [13],[14] and inflammatory markers [15], which are commonly connected to adverse health effects. Moreover, the effects of stress during adolescence can remain latent until they manifest in adulthood [16], highlighting the importance of researching and better understanding how to intervene effectively during this stage to prevent long-term negative consequences.

While neuroscience has traditionally focused on the internal mechanisms of cognition and behavior, in the last years, there has been an increasing focus on the influence that social relationships and experience have in shaping such mechanisms [5]. Socioneuroscience was created to provide theory and evidence to fully understand how social relations influence brain architecture and functioning, development, and plasticity [17]. As an interdisciplinary field that bidirectionally bridges natural sciences and all social sciences, socioneuroscience has deepened the understanding of how different types of social interaction and relationships impact brain, behavior, and health in different ways [18]. As a key contribution, based on empirical evidence of the impact of the coercive dominant discourse on many individuals' attraction and election of sexual-affective relationships [19], it has theorized on the impact of the coercive dominant discourse on human volition, suggesting that it leads to enslaved desires and memories and, thus, to imprisoning many individuals' sexual freedom [17]. Most importantly, socioneuroscience also indicates that, thanks to brain plasticity, actions and interventions that challenge the coercive discourse and break the imposed link between attractiveness and violence, rendering egalitarian behaviors as attractive, contribute to changing many individuals' attraction and desire, which can reverse the aforementioned negative consequences of violent relationships. In this way, such actions potentially provide individuals the opportunity to break free from the neural wiring that the coercive discourse imposes, contributing to overcoming violence and its consequences.

In this regard, research has shown that the implementation of the Zero Violence Brave Club (ZVBC) has not only helped prevent violent relationships but has also strengthened the protection of children while improving their health and well-being [20],[21]. The ZVBC is a “successful action (SA) that overcomes any kind of violence in all types of contexts. It is composed of the members of one community or institution which announces them as the ones always supporting any member that is suffering or feels scared to suffer any kind of violence, acting as a shield that guarantees the protection of the victim and their transformation into a survivor” [22]. Although it originated in schools, it is implemented in very different contexts, not only educational ones, and the impacts of its rigorous implementation have been reported in very different contexts with participants of different ages and backgrounds [23]. It is grounded on the theory and research line on preventive socialization of gender violence, which analyzes the processes that socialize individuals and groups in sexual-affective relationships, identifying a coercive dominant discourse that gives visibility and attractiveness to violent behaviors and relationships [24]. Given that many adults are usually unaware of the violence affecting children, the ZVBC encourages them to be the ones who break the silence and act to protect an actual or potential victim. A key for the success of the ZVBC in promoting 0 violence spaces is that it links the language of desire to the language of ethics, that is, removing attractiveness and protagonism from people who use violence and portraying upstanders as attractive and admired due to the braveness they demonstrate by standing up with victims. In this way, more and more students want to be upstanders, and violent behaviors are seen as ridiculous and undesirable, leading to a decrease in violence and isolating violence against those who defend victims. In the ZVBC, students know they cannot leave any victim alone, that they have to protect anyone who is suffering and those who defend them, and that reporting violence does not mean being a telltale but being brave.

The extensive scientific literature on the adverse effects of toxic relationships on the brain and health is essential for increasing awareness on the urgency of promoting spaces free of violence for all, especially for the youngest ones. However, more evidence on the positive impacts of the actions that overcome and prevent violence is needed. This qualitative case study advances in this direction by analyzing a teacher's perception of the impact of the ZVBC implemented in his second-grade primary school classroom. The goal is to identify and analyze the teacher's perceptions of the impacts of the ZVBC among his students' wellbeing. It is expected that the qualitative evidence presented in this article will be used in future research to demonstrate the ZVBC's impacts in student's architecture and functioning of the brain.

## 2. Materials and methods

In order to achieve the objective set in this study, researchers posed the following research question:

What are a teacher's insights, perceptions, and reflections on the impact of the ZVBC among his students' wellbeing during the period of its implementation in his classroom?

To respond to this question, the teacher's audio-diary with a periodic reporting on students' interactions, relationships, and behaviors throughout the 5-month implementation of the ZVBC is analyzed. The field diary methodology is complemented with an interview to the teacher at the end of the implementation of the ZVBC.

The methodology follows the communicative approach [25], which has been taken up by the European Commission as co-creation; now a requirement for all research projects funded by it in order to achieve social impact [26]. Within this framework, in this case, the qualitative case study design has enabled researchers to gain in-depth insights of the teacher's experience implementing the ZVBC in his classroom. While this design provides researchers affordances such as a detailed exploration of the process and evolution of the ZVBC through the teacher's eyes and voice, it is not without limitations. The main limitation is that no causality can be drawn between the implementation of the ZVBC and the improvements the teacher has observed among his students; this study is limited to analyzing the teacher's interpretations of the relation between implementing the ZVBC and the improvements he observes among his students.

### 2.1. Participants and school context

Participants are a male second-grade teacher of primary education school, and the students that compose his classroom include 23 children (10 boys and 13 girls), aged 7 and 8 years old. The school is in a medium-sized town in a southern European country, targeting a population of middle-class families. Students had never participated in the ZVBC before their teacher implemented it.

### 2.2. The ZVBC intervention

The teacher implemented the ZVBC in his classroom from January to June. The main reason why he implemented it, and why this intervention has been selected as the focus of this study, is that the ZVBC was identified in the NESET report on overcoming violence against children as one of the 13 interventions successful in overcoming and preventing violence, and among those 13, it is the one on which most impacts have been reported [21].

He did it following several steps, some of them a requirement of the ZVBC for its successful implementation and others based on his own criteria. First, on the first day of implementing the ZVBC, the teacher explained what it is (i.e., brave boys and girls are upstanders, who stand with victims of violence). Second, the students engaged in dialogue among each other and with the teacher and agreed on the attitudes and behaviors they would and would not accept among themselves, both in the school and outside. In this way, the students co-created with the teacher the rules they would have to follow in order to be part of the ZVBC. Third, they reached a consensus that if any of them performed in a way the group did not accept (according to prior agreement), the student would be left out of the ZVBC, and they would all talk about what happened. Throughout the dialogue, as well as whenever violence occurred throughout those five months, the teacher reminded students of the “0 violence” criterion, that is, that no violent act would ever be justified, regardless of how small such an act could seem. The next step was implemented whenever a student or several students reported someone had treated another one in a way the classroom decided was not allowed. The teacher would ask the students to explain what happened, giving the floor to anyone who would like to comment on it or tell the person who behaved with violence that they did not allow such behaviors. If the behavior was not accepted among the rules the students had created for the ZVBC, the student who did it would be left out of the ZVBC for the rest of the day. In this way, attention is put on the victim and his/her supporters, not on the bully, from which attention is completely removed [19],[27].

The expulsion from the ZVBC is carried out symbolically by setting aside the photo or the name of the student from the rest of the class. When implementing the ZVBC, it is made clear that everyone, without exception, can be brave, since the focus is on actions and behaviors, not on people. When someone leaves the ZVBC for a day, they know that if they stop showing that type of behavior and replace it with brave attitudes, the group will value them very positively [28]. Every morning, the whole class is once again in the ZVBC.

### 2.3. Data collection

On the one hand, a communicative observation was conducted by the teacher during the five months of the implementation of the ZVBC in the classroom. Through a field diary methodology, the teacher periodically recorded audios accounting his interpretations of children's interactions, behaviors, and attitudes. Throughout those months, the teacher met with some of his students' parents and included reflections from those meetings in his audio-diary as well. The teacher recorded the audio recordings on his phone whenever: 1) Violent acts against any of his students were reported to him, 2) students who usually behaved with violence changed such behaviors, and 3) he interpreted students felt better physically or emotionally. In all, he recorded 23 audio recordings that spanned 1 to 13 minutes.

We analyzed the audios in light of scientific evidence and theory on the preventive socialization of gender violence and on the ZVBC. Following the communicative approach, they engaged in an egalitarian dialogue with the teacher in order to share their interpretations of the data with him and complement the analysis with his insights. In this semi-structured interview conducted in June, we asked the teacher to expand on his perceptions of students' wellbeing improvements thanks to the ZVBC, as well as to provide more details on how it worked in specific episodes reported in his audios. The interview was also audio-recorded.

This follow-up was conducted by the teacher during the two weeks prior to the implementation of the ZVBC and the three weeks afterward.

### 2.4. Data analysis

Data analysis involved two qualitative analytical levels. On the one hand, after transcribing the audio recordings of the communicative observations and the interview, the teacher's accounts of all interactions regarding relationships among students and behaviors toward each other were identified. All these instances were then analyzed more in depth in light of the theory and scientific evidence of communicative acts. This framework considers all signs of human communication when studying how we construct reality, relationships, and identities in our communication, such as words, tone, gaze, caresses, body language, smell, emotions, feelings, likeness, social status and power position, intentions, desires, or consequences [29]. In this study, when analyzing the teacher's accounts of students' behaviors, interactions and relationships, we used the concepts of *power communicative acts* and *dialogic communicative acts* as the two major categories for the first analytical level. Power communicative acts are all those signs of communication that are used to seek action through coercion and pressure, deceit, imposition, or violence, whereas dialogic communicative acts are those based on honesty, respect, solidarity, and a desire to achieve action through consensus and freedom and equality [30]. The former category included all interactions showing power relations and interactions, such as using physical, verbal, or symbolic violence or engaging in non-consensual relationships. Under the latter category, all interactions showing dialogic relations and interactions were included, such as supporting and defending victims and helping each other.

The second analytical level involved analyzing the teacher's perceptions of the consequences of the power communicative acts and dialogic communicative acts identified in the first step. Under this frame, two thematic categories were inductively created and analyzed: Negative and positive consequences. The former category included consequences such as crying before going to school or decreasing participation in class. The latter category included consequences such as increased motivation, happiness, and well-being, among others. The analysis categories and procedure are summarized in Table 1.

**Table 1.** Analysis categories.

**Procedure**	**Category**	**Example**
Analytical level 1	Power communicative act	A student insults another one
	Dialogic communicative act	A student tells the teacher other students are hurting one of his classmates
Analytical level 2	Negative consequences of violence in the classroom	A student says his stomach hurts whenever older students threaten him
	Positive consequences of decrease of violence in the classroom	A student starts participating more in class and wanting to go to school after violent behaviors decrease

To ensure inter-coder reliability, two researchers conducted the analysis. First, they did so individually, manually coding each interaction that fit any of the aforementioned categories. Once a preliminary categorization was made, the three researchers compared their analyses, engaging in dialogue whenever there were different categorizations in order to reach a final agreement.

### 2.5. Ethics approval of research data collection

This study was conducted following international guidelines and requirements, such as the principles of the Universal Declaration of Human Rights and the EU's Charter of Fundamental Rights (CFREU). The parents of all students participating in the study signed informed written consent, which included information on the anonymization and storage of the data collected, and the purpose and methodology of the study. The study received approval by the Ethics Committee of the Community of Research on Excellence for All, with approval number 20250612, and of the University of Barcelona, with approval number 332.25.

Because the study involves sensitive events, strict confidentiality procedures were implemented. All data were handled exclusively by two members of the research team and stored in secure, encrypted files with restricted access. It was also ensured that no student could be identified. All these measures were strictly applied to guarantee the highest level of protection for underage participants.

## 3. Results

The results section is divided into two major subsections based on the two major categories from the second analytical level: The teacher's perceptions on the negative consequences of power communicative acts among students and of the positive consequences of dialogue communicative acts. While our objective of the study was to analyze the teacher's insights and reflections on what he considers positive impacts of the ZVBC among his students' wellbeing, some of the negative consequences of violence he perceived before implementing the ZVBC are included in order to better understand the changes he considers have occurred with the intervention.

### 3.1. Teacher's perceptions on negative consequences of violence among students

The teacher, Francesco, stated that prior to the implementation of the ZVBC, there were serious violence cases in the classroom, including a student, Luca, who had thrown another one, Alessandro, down the stairs, which led to the opening of the anti-bullying protocol in the school. Luca had been bullying his classmates since kindergarten:

When I started [working in this classroom], I saw how the situation was, well, complicated, because Luca, despite having a teacher watching him 24/7, constantly made his classmates cry.

As the teacher noted, before implementing the ZVBC, it was common for students to cry in class due to this student's physical and/or verbal aggressions. Indeed, Francesco explained that this classroom was known in the school due to its high rate of conflicts and violence, creating an atmosphere full of toxic relationships. Many students did not to want to go to class, as is the case of Alessandro, the student whom Luca threw down the stairs:

Before, until Easter, to come to school, they had to push him in. He didn't want to come to school. Last year, in 1st grade, and this year, at the beginning too. And he said, well, he didn't say much, but when he started to explode and explain things, they saw that he was suffering a lot.

Even though Alessandro did not explain much of the violence he was a victim of, Francesco could perceive he was suffering. It was similar to the case of Fabrizio. Fabrizio's mother explained to Francesco that, before the ZVBC, since her child was in kindergarten, he would cry every week before going to school:

She told me that when they were in kindergarten at 3 and 4 years old, her son Fabrizio cried every Sunday saying that he didn't want to go to school the next day because of Luca.

Even students like Isabella, who used to have a very high motivation toward learning and participating in school, started losing the spark due to the adverse classroom environment over the last years:

She has always had this motivation to learn, but [her parents] saw that before I arrived, that this motivation and desire to come to school was fading. The mother said that she could see that there were still some sparks of desire to learn that were fading away. She used to say that the class atmosphere was very bad. “There were problems all the time. People didn't respect each other's turn.” So, she explained to her parents why she decided to stay passive in class, do her work, and not get involved in anything.

In the midst of this generalized violent atmosphere, there was a serious case of sexual assault according to Francesco. One of the students had been sexually assaulted by Luca. He had touched her intimate parts in the bathroom and, as Francesco interpreted it, had her completely subordinated. As Francesco explained, “when I asked her something in math or language class, whenever she started to speak, she would look at Luca — he was the one who had her under his control”. Not only did she not dare to participate in class and was dependent on Luca's attitude toward her, but she explained the harassment as positive:

When I first started, Alice said that Luca was her boyfriend and that they had kissed. When she talked about him touching her in the toilet, she said it while laughing, as if it were something funny.

### 3.2. Teacher's perceptions on the positive consequences of the ZVBC among students

From the very first week in which the ZVBC was implemented until the end of the school year, Francesco reported changes in students' relationships within and outside the classroom. As stated by the teacher, physical violence among his students decreased immediately after implementing the ZVBC, and the former adverse relationships soon transformed. The teacher was not the only one who noticed an improvement in the classroom. The girl doing her practicum in this classroom told him how amazed she was at the solidarity among students and the positive classroom climate she witnessed:

On Monday, a girl started her practicum, and she is delighted with the way children help each other. She is fascinated to see such a good atmosphere, to see that they are at ease in class. And when they see that someone needs help, they are willing to help. And the truth is that, well, the atmosphere is much better than when I started. Besides the fact that there is no violence anymore, you can see that when it comes to work, they help each other more, they are calmer, they are more motivated.

As he indicates, bullying and other forms of violence disappeared, but this was not the only evident change: The prevailing violent relationships and the absence of caring and responsive relationships were replaced by solidarity, mutual help and, in some cases, friendship and caring relationships. Even in the case of the anti-bullying protocol, 2 weeks after starting its implementation, the principal and counselor decided to loosen the measures marked by it, as they had observed a lack of violence during those weeks:

On Tuesday, I met with school management and counseling because we agreed to talk that day about the bullying protocol. And when I told them about the situation, they told me that we were going to loosen the measures (...). So, now it is clear that with these changes, it is no longer necessary to have a teacher watching them all the time, and the measures will be loosened.

From constantly making his classmates cry, even with a teacher watching him at every minute a few weeks earlier, not only did Luca stop his violent and abusive behavior toward Alessandro, but he also started defending him when students from other classes insulted him, as Francesco recalled:

When Luca defended him, Alessandro came to tell me about it. “Luca defended me, and he told Enea, the guy in the other class, that I didn't like it.” And, well, we talked about it in class and the whole class backed Luca and it was, I mean, Luca's face was amazing, he really looked very happy. Also, since Luca had stood up for Alessandro, he realized that, of course, the whole class was also saying nice things to him.

Francesco saw that Luca became an upstander and, while everyone was afraid of him before, since his participation in the ZVBC, his classmates started to value him due to his brave attitude when defending Alessandro. This was a significant moment for Luca to see that his violent behavior no longer had the desired effect on his classmates, who now only valued him when he had upstander behaviors. His behavior changes not only led to a change in how others viewed him, but also how they related with him, even defending him when older students started insulting him:

Two of the fifth-grade students started to tease Luca, saying: “Kid, you're crazy” and things like that. And 3 [classmates], who are three years younger than the fifth graders, confronted them and they told them no, that they didn't like that. In class, when talking about the case, Luca started to cry, but out of emotion. And I asked him, “Do you want to say something?” And he said: “I want to thank them for defending me” crying, but like crying his eyes out.

Highlighting the bravery of students who defend victims led to many of them wanting to help and defend others. As Francesco recorded in his diary a few weeks after implementing the ZVBC, “today, the kids came wanting to defend someone”. Yet he thinks students' wish to stand up against violence and be recognized as upstanders started much earlier, as early as in the second day in which the ZVBC was implemented:

I should point out that the first day we did the Zero Violence Brave Club, when Luca came out of the club, only 3 people talked to tell him that they didn't like what he had done and I, as a teacher, told those 3 people, I repeated their names 4 or 5 times, saying that those 3 people were very brave, taking a stand for the victim and telling Luca that they didn't like what he had done. When I said that these 3 people were very brave, the faces of the rest of the classmates changed. And on Friday, when Luca came out of the club again, the people who spoke to tell him that they didn't like what he had done before were, instead of 3, almost the whole class, maybe 18–19 people out of 23.

In only two days after implementing the ZVBC, almost the whole class broke the silence against Luca, telling him, to his face, they did not like his violent and toxic behaviors. Eventually, even students who used to remain passive in front of violence became some of the most active ones in defending victims, not only in the classroom itself, but in any context:

Today, when one of the boys who stood up in the park stayed with the victim while the others went to get help, on the way home, the boy (Leonardo) said to me, “Francesco, so I was an upstander, right?”

As Francesco states, it is not the case of 1 or 3 students, but it is the whole classroom that became an upstander within and outside its walls:

They get together to defend themselves, to hold together at the moment and form a mini-club to defend someone who has been attacked. And, well, it's amazing how they talk about it, how they feel that people's problems are their own, how they get involved in defending their classmates and, well, and I've told them, thanks to that, we are very happy in class and with such a good atmosphere.

Francesco also identified that the decrease in violence and the increase of solidary relationships among students made many of them become more motivated to go to class and learn. According to the teacher's perception, such a classroom climate was replaced by a more supportive, motivating, and bully-free environment.

During meetings with several students' parents, all of them expressed gratitude to the ZVBC due to the improvements they saw in their children's mood, happiness, and well-being, as stated by the teacher. For instance, in the case of Alessandro:

The father said that he saw that Alessandro has a different attitude. (...) he says that, this year, he wants to come to school on his own. And that he is very happy to come.

In a different meeting, Alessandro's mother expressed similar feelings:

I took the opportunity to ask her [the mother] how Alessandro was doing and she told me that he was doing very well and that he came to class happy and eager, when before Christmas, they were here [at the school] every week, worried that their son was being bullied. So, it seems that in these 3 or 4 weeks, we have reversed the situation, and the family sees their son going to class happy.

Fabrizio's mother also showed relief and happiness seeing how happy and motivated her son was to go to school every day. She even stated she was surprised when she learned that her son was now sitting next to Luca:

In the meeting, Fabrizio's mother told me that he really enjoys the [Zero Violence] Brave Club and that he's very motivated. She said he's very eager to come to class, that he feels really comfortable with me, and that ever since I've been here and since we started the club, everything has been great. The other day, even though he had a fever, he still wanted to go to class. Today, she found out that her son sits next to Luca and was a bit surprised.

Similarly, Isabella's parents saw a change in her motivation to come to class and participate, according to Francesco:

Her parents said that now, yes, they see her motivated and that she talks about many of the things we discuss in class. They see that she feels comfortable and engaged, something that wasn't the case before January. Back then, she was struggling to come to school and didn't want to attend.

According to the teacher's perception, in addition to improvements in students' mood, motivation, and well-being, he also noticed an overall decrease in gastrointestinal symptoms that had students reported.

In general, stomach aches and headaches have significantly decreased. It's true that in spaces where there's no ZVBC, like the cafeteria, they complain more and sometimes come in the afternoon saying their stomach or head hurts. But in class, it's completely different. When I started, there was an environment where violence was very normalized, and it was very common for a child to come to me saying their stomach or belly hurt. One day, a girl told me her belly hurt, and I asked her what she had in the afternoon. At first, she didn't want to tell me, but then she said she had swimming lessons after school and didn't want to go. Another day, the same thing happened with another girl who didn't want to go to her extracurricular activity. I don't know to what extent they really feel that pain or if they make it up to avoid going to a place where they don't feel comfortable, but it's clear to me that when there is widespread mistreatment, complaints about stomach or head pain are much more frequent than when they are treated well and the environment is positive.

Regarding the sexual harassment case, Francesco also identified a change in Alicia's narrative about it, observing a lack of comments referring to Luca as her boyfriend or to the harassment as kisses:

In a more indirect way, those kinds of comments have disappeared. It's been quite a while since she's said anything like “I kissed this boy”, and, of course, she hasn't mentioned anything about the toilet incident again. There is also a change in her attitude when we talk about respecting each person's body, for instance in the bathroom. Now she remains silent, whereas before the ZVBC, she would always talk about what happened to her laughing.

He also noticed her dependence of Luca had disappeared when participating in class, which now she did often, freely, and with great motivation:

The [Zero Violence] Brave Club has made a huge difference for her. (...) Now things have changed, the atmosphere has changed, and she really enjoys participating. She genuinely tries to answer questions, both in math and language class.

Francesco interprets that this split in the association between violence and attractiveness, which is now put in brave people who stand up against violence and defend victims, also changed Luca's behavior:

While the class was telling Giorgia how brave she had been, Luca's expression changed. That was on Thursday, and it was the first day he didn't leave the Brave Club. So, it's quite possible that he realized something, that being brave, like Giorgia who stood up for Adriano, is starting to be seen as something valuable and admirable.

Francesco could see in Luca's expression a realization that his classmates started valuing people who defended victims as attractive. Whether by coincidence or not, it was the first day in which he did not behave with violence. A few weeks later, not only did Luca stop behaving with violence, but he started defending his classmates, even the ones he had previously attacked:

Luca stood up for Alessandro when others were calling him names and teasing him. It all started during a game of tag, when someone gets caught, they say it's Alessandro, but apparently, they keep teasing him even after the game ends, in the toilet, and on the stairs. Luca defended him and told the others, “I don't like it when you call him that.”

Francesco also perceived that Luca's mother, who was previously concerned with his well-being and mental health due to his violent behaviors, became relieved when seeing his behavior changed, associating being included and respected when behaving without violence and defending victims:

[before the ZVBC] his mother, although she wants to take action, feels that her son won't truly be happy if he continues relating to others in such a violent way. [during the ZVBC] now she sees that her son is really engaged with the Brave Club. I also told her in a message that he hasn't left the club at all this week. She replied that she already knew, every day when he gets home, he tells her he stayed in the club. She says he seems very excited about it, and she also added that, in general, she sees him happier, more content, and more at ease.

During the first week in which Luca was not removed from the ZVBC for the whole week, both Francesco and Luca's mother noticed he was hooked to the ZVBC.

Last, Francesco has observed changes among many students when it comes to learning and class participation. One of the clearest cases of this change for Francesco was that of Alice, the girl who had been sexually harassed:

Alice had been identified as having special educational needs and was receiving five sessions per week with the therapeutic pedagogy teacher. The thing is, she didn't understand anything she read, absolutely nothing. And in math, she couldn't even do 8 + 2. It was quite incredible. (...) But now, she no longer needs therapeutic pedagogy support. Since the [Zero Violence] Brave Club started, her attitude toward learning has completely changed. Her attention span and memory have improved significantly. She's much more able to concentrate on her work and is learning so much more. The therapeutic pedagogy teacher was amazed by the change.

According to Francesco, the girl was no longer considered by the teachers as a student with SEN who needed help with everything. Both him and the therapeutic pedagogy teacher have seen a change in her attention capacity, stating she is now much more able to concentrate and learn. Along this line, he expressed that:

Her memory and attention capacity, which once seemed impossible to improve, especially since she had epileptic seizures in early childhood, have shown remarkable progress. It used to seem like those challenges would prevent her from ever improving in those areas. But thanks to the [Zero Violence] Brave Club and other successful educational actions, it's become clear that she can concentrate, and she can learn and retain what she's learning. (...) One clear impact is that we recently did reading comprehension tests in both the medium of instruction and another curricular language subject. In the second one, she answered 6 out of 12 comprehension questions correctly — more than many of her classmates. And it was a difficult text with complex questions, so it's not just a matter of guessing.

Not only did Francesco perceive an improvement when seeing her classroom behavior, being more concentrated and participating more, but also in her test results. Similarly, Giovanni, who had difficulties speaking in the medium of instruction, started learning and developing this language communication skill thanks to wanting to be acknowledged as a brave person. According to Francesco:

Children who previously didn't speak or share anything are now opening up a lot. For example, Giovanni, who has difficulties speaking the medium of instruction, has actually started speaking more, especially when it comes to reporting things he sees. And it's because he feels that speaking up is valued. He wants me to tell the whole class that he spoke up and that he was brave.

## 4. Discussion

There is an extensive body of literature on the negative brain and health consequences caused by different factors in human beings' environments. Socioneuroscience has provided key contributions that enable the advancement toward a more robust understanding on the influence of social relations in the architecture and functioning of the brain. However, most researchers have focused on the negative alterations in the brain related to violent and coercive relationships [16],[17]. This study opens a pathway that aims at providing scientific evidence on the impacts of actions that overcome violence in the architecture and functioning of the brain. To advance in this direction, we have provided qualitative scientific evidence from a teacher's perceptions on the positive impacts of the ZVBC among his students' wellbeing. Such evidence, when linked to existing and future evidence from neuroscience and socioneuroscience, can help better understand the neural consequences of overcoming violence.

Research has shown that adverse social relationships are related to toxic stress, negative thoughts, and chronic stress [6]. In the classroom analyzed, prior to implementing the ZVBC, adverse relationships prevailed, which could lead to the assumption that students from this classroom, many of whom cried every day in school, had toxic stress. Another related and common symptom identified in the literature on negative consequences of adverse relationships that include violence is a deterioration in students' mental health. The teacher identified mood changes among some of his students, not only at school, but also at home, suffering and crying before going to school. According to the teacher, since the ZVBC, students no longer had such negative thoughts. In light of evidence on the consequences of violence in students' mental health, the perceived improvements in students' wellbeing could indicate a potential reversal in students' epigenome and telomeres caused by stress [3],[4],[6]. Although it remains unknown what impact the implementation of the ZVBC during these five months will have on students in the long term, at least during those five months, students have been free from violence in their classroom and, therefore, free from the negative consequences of violence. This could have important implications in weakening and reversing the impact of toxic stress on the hypothalamic-pituitary-adrenal axis [9]–[11] or on increasing inflammatory markers [13],[14], among others.

Another symptom associated with toxic relations are gastrointestinal symptoms, commonly found related to early life adversity [8]. Francesco found a significant decrease in students' reporting of such symptoms: Whereas it was common for students to complain about stomachaches before the implementation of the ZVBC, he saw a sudden decrease in such complaints once the classroom was free of violence and there was an overall positive and solidary environment. The only moments in which students complained of such symptoms once the ZVBC started to be implemented were when students came from other school spaces in which there had been conflicts or were going to extracurricular activities with adverse environments.

Much research has demonstrated alterations in neural architecture and functioning, such as: Alterations in learning, memory, and executive functioning due to toxic social experiences [11], weakened brain development, and architecture related to environments that contain relationships and contexts that involve violence, shrunk brains, and neural alterations associated with an absence of caring and responsive relationships [22], or alterations in brain architecture and brain functioning due to abuse and neglect [23]. The positive changes in students perceived by the teacher could potentially decrease or weaken such negative consequences and foster a positive and healthy brain architecture in the students.

Indeed, the teacher has provided examples of students who, prior to the implementation of the ZVBC, had difficulties speaking the medium of instruction, or to concentrate and understand simple sentences. These examples, according to the teacher, show the contrast between the toxic classroom environment prior to the intervention and the more supportive atmosphere observed afterward. This change, following research on the negative impacts of violence on students' memory and cognitive functioning, could help prevent alterations in the neural architecture of the amygdala, hippocampus, and prefrontal cortex that are often associated with violent and toxic contexts [1],[2]. The case that best illustrates this is that of Alice. Francesco comments that, based on his classroom observations and seeing Alice's change in attitude, some of the symptoms she displayed (such as those associated with neural alterations in learning, memory, and executive functioning) may have been aggravated by the sexual harassment she had suffered. Moreover, it seems the school associated her weakened brain development and architecture with the epileptic seizures she suffered in her early childhood. However, Francesco saw a clear improvement in her learning, memory, and executive functioning, as well as her ability to concentrate when the harassment disappeared, seeing her capable of concentrating and of understanding and retaining school content. The results she obtained in the reading comprehension tests appear to corroborate these impacts.

Last, the scientific literature has also indicated neural consequences related to the coercive dominant discourse that promotes and strengthens violence. These consequences include the internalization of the association between attractiveness and violence, shaping individuals' cognitive and affective schemata, and controlling their cognitive and emotional interpretation of autobiographical memories of violent sexual-affective relationships with feelings of attraction [16],[17]. With the very case of Alice, Francesco saw a transformation in her subjugation to her former harasser and in her narratives about the harassment. On the one hand, while Francesco saw that Alice would not dare to speak in class without having Luca's approval, since the ZVBC, she stopped calling him her boyfriend and started participating in class with more confidence. On the other hand, before the ZVBC, her narratives of the sexual harassment seemed to be influenced by the coercive dominant discourse, which could indicate that her cognitive and emotional interpretation of her autobiographical memories related to the harassment were positive. This can be seen when Francesco said she called Luca her boyfriend or when she laughed when recounting the harassment. Looking at Francesco's account of the impact of the ZVBC, it appears Alice's cognitive and affective schemata changed from the way in which she previously associated violence with fun or attractive. As he states, when issues on consent or harassment come up in class, she no longer says anything, as she did before. Along this line, the cognitive and affective schemata also appear transformed in Luca himself, as, according to Francesco, he stopped behaving violently when he realized his classmates rejected such attitudes and valued brave attitudes as the attractive ones.

## 5. Conclusions

Scientific evidence on the brain and health consequences of violence is necessary in order to increase awareness of the grave, often lifelong consequences of violence, and to create pathways to overcome and prevent it in all spaces. However, citizens and science are increasingly demanding scientific evidence of social impacts, that is, scientific evidence of actions or interventions that achieve improvements in citizens' marked goals, such as Sustainable Development Goals. Socioneuroscience engages evidence from all natural and all social sciences in dialogues in order to better and more deeply understand the impacts of social relations and interactions on individuals' brain architecture and functioning. This study takes a step in this direction by providing qualitative evidence on a teachers' perceptions of the impact of the ZVBC in improving his students' wellbeing.

It is important to acknowledge limitations of the study. The article presents perceptions of a teacher on the impacts of the ZVBC on his students. Such perceptions should be contrasted with more individuals in students' environments, as well as with the students themselves. Moreover, the data collected for this study does not include brain and health indicators. Future research should contrast the qualitative evidence provided in this study with evidence from neuroscience to identify and analyze the neural architecture and functioning consequences of the ZVBC.

## Use of AI tools declaration

The authors declare they have not used Artificial Intelligence (AI) tools in the creation of this article.
